# Mixing of moiré-surface and bulk states in graphite

**DOI:** 10.1038/s41586-023-06264-5

**Published:** 2023-07-19

**Authors:** Ciaran Mullan, Sergey Slizovskiy, Jun Yin, Ziwei Wang, Qian Yang, Shuigang Xu, Yaping Yang, Benjamin A. Piot, Sheng Hu, Takashi Taniguchi, Kenji Watanabe, Kostya S. Novoselov, A. K. Geim, Vladimir I. Fal’ko, Artem Mishchenko

**Affiliations:** 1 Department of Physics and Astronomy, University of Manchester, Manchester, UK; 2 National Graphene Institute, University of Manchester, Manchester, UK; 3 State Key Laboratory of Mechanics and Control for Aerospace Structures, Key Laboratory for Intelligent Nano Materials and Devices of Ministry of Education, Institute for Frontier Science, Nanjing University of Aeronautics and Astronautics, Nanjing, China; 4 Key Laboratory for Quantum Materials of Zhejiang Province, Department of Physics, School of Science, Westlake University, Hangzhou, China; 5 Laboratoire National des Champs Magnétiques Intenses (LNCMI), CNRS Université Grenoble Alpes, Université Toulouse 3, INSA Toulouse, EMFL, Grenoble, France; 6 State Key Laboratory of Physical Chemistry of Solid Surfaces, Collaborative Innovation Center of Chemistry for Energy Materials (iChEM), College of Chemistry and Chemical Engineering, Xiamen University, Xiamen, China; 7 National Institute for Materials Science, Tsukuba, Japan; 8 Institute for Functional Intelligent Materials, National University of Singapore, Singapore, Singapore; 9 Henry Royce Institute for Advanced Materials, Manchester, UK

**Keywords:** Electronic properties and materials, Surfaces, interfaces and thin films

## Abstract

Van der Waals assembly enables the design of electronic states in two-dimensional (2D) materials, often by superimposing a long-wavelength periodic potential on a crystal lattice using moiré superlattices^1–9^. This twistronics approach has resulted in numerous previously undescribed physics, including strong correlations and superconductivity in twisted bilayer graphene^10–12^, resonant excitons, charge ordering and Wigner crystallization in transition-metal chalcogenide moiré structures^13–18^ and Hofstadter’s butterfly spectra and Brown–Zak quantum oscillations in graphene superlattices^19–22^. Moreover, twistronics has been used to modify near-surface states at the interface between van der Waals crystals^23,24^. Here we show that electronic states in three-dimensional (3D) crystals such as graphite can be tuned by a superlattice potential occurring at the interface with another crystal—namely, crystallographically aligned hexagonal boron nitride. This alignment results in several Lifshitz transitions and Brown–Zak oscillations arising from near-surface states, whereas, in high magnetic fields, fractal states of Hofstadter’s butterfly draw deep into the bulk of graphite. Our work shows a way in which 3D spectra can be controlled using the approach of 2D twistronics.

## Abstract (web summary)

The electronic states in three-dimensional crystals such as graphite can be tuned by a superlattice potential occurring at the interface with crystallographically aligned hexagonal boron nitride.

## Main

At the surface of a crystal, its periodic lattice is interrupted, and surface states arise with wavefunctions exponentially decaying into the bulk of the crystal^25^. For example, surface charge accumulation in semiconductors leads to distinct 2D subbands tunable by electrostatic gating. By contrast, in metals, the high charge-carrier density makes it difficult to observe and control surface states, as the bulk shunts the surface conductivity. Lying in between these two extremes are semimetals such as bismuth and graphite, which have tunable surface states that are interesting but remain underexplored. Graphite films are of interest as they show both 3D and 2D electronic properties controlled by electrical doping and an external magnetic field *B*. Notably, graphite of a finite thickness exhibits an unusual 2.5-dimensional (2.5D) quantum Hall effect (QHE)^26^.

**Fig. 1:**
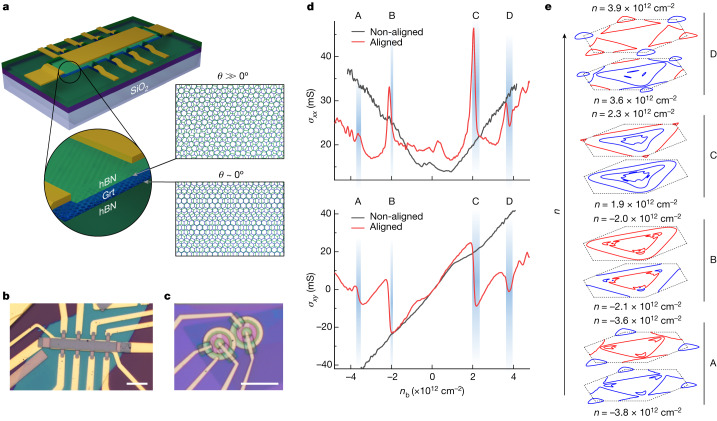
Moiré superlattice at the graphite–hBN interface. **a**, Schematic of a heterostructure device with graphite (labelled Grt) encapsulated in hBN with one of the interfaces aligned. Here the lattice mismatch between graphite and hBN has been exaggerated for clarity. **b**,**c**, Optical micrographs of devices D1 (**b**) and D3 (**c**). Scale bar, 10 μm (**b** and **c**). **d**, Conductivities *σ*_*xx*_ and *σ*_*xy*_ as a function of the carrier density induced by the bottom gate, *n*_b_, for aligned device D1 and non-aligned device D4, measured at *T* = 0.24 K and non-quantizing *B* = 120 mT. **e**, Line cuts through the calculated dispersion relation in the *k*_*x*_–*k*_*y*_ plane of the SBZ, at carrier densities (bottom to top) *n* (×10^12^ cm^−2^) = −3.8, −3.6, −2.1, −2.0, 1.9, 2.3, 3.6 and 3.9, grouped as pairs. Labels A, B, C and D correspond to the regions highlighted in **d**. The black dashed hexagon denotes the boundary of the first SBZ and red curves denote the hole and blue curves denote electron Fermi-surface cuts. Some lines at the corners are extended into the second SBZ for clarity.

In this Article, we explore moiré engineering of highly tunable electronic states, by aligning two bulk crystals, hexagonal graphite and hexagonal boron nitride (hBN). To this end, we prepared hBN/graphite/hBN heterostructures by aligning thin graphite films (about 5–10 nm thick) on top of the hBN substrate and encapsulating the stack with another hBN crystal. Unless otherwise stated, this latter, encapsulating, hBN is intentionally misaligned (see Methods, ‘Device fabrication’ for details). As the lattice constants of hBN and graphite are close, in the heterostack, they form a moiré superlattice with the periodicity controlled by the lattice mismatch, *δ* = 1.8%, and a misalignment angle, *θ* (Fig. 1a). In addition to providing the moiré superlattice, the hBN encapsulation also preserves the high electronic quality of graphite films^26–28^. Figure 1a–c shows schematics and micrographs of the hBN/graphite/hBN heterostructures, fabricated into Hall bar and Corbino geometry devices. In these devices, the top and bottom electrostatic gates were used to independently control carrier densities *n*_t_ and *n*_b_, at the top and bottom interfaces of the hBN/graphite/hBN heterostructure. In total, we have studied 11 graphite heterostructure devices (Extended Data Table 1).

**Extended Data Fig. 1:**
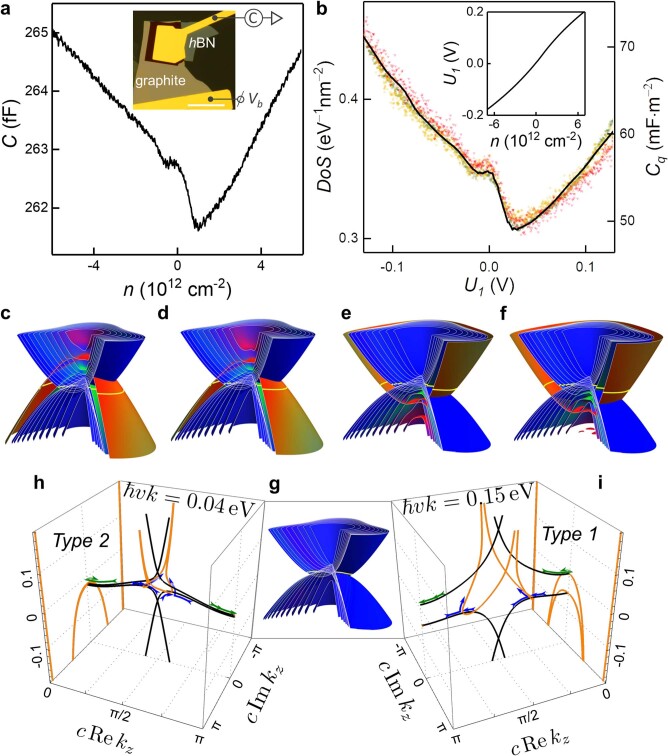
*DoS* spectroscopy of surface states of graphite in zero magnetic field. **a**, Capacitance (*C*) *vs* carrier density (*n*) for device D6 at *T* = 0.3 K. Inset shows optical micrograph of a typical capacitor device, scale bar 20 µm. **b**, Density of states (*DoS*) and quantum capacitance (*C*_*q*_) *vs* surface potential (*U*_1_) from calculations based on effective-mass model (black line). Coloured symbols are experimental data from four different devices (D5, D6, D8, D11). Inset is calculated *U*_1_ *vs n*. **c–f**, Calculated dispersions of a 20-layer-thick graphite film for hole/electron dopings *n* = −6, −4, 4, 6 × 10^12^ cm^−2^ as a function of in-plane momentum *k*_*x*_,*k*_*y*_ (horizontal axes) and energy *E* (vertical axis). Red (green) colour indicates surface states having high probability density of the wavefunction at the first (second) graphene bilayer. Upper outer surfaces with larger radius correspond to Type 1, and lower inner surface with a smaller radius correspond to Type 2. Blue colour indicates bulk states, and yellow contour highlights the Fermi level. **g**, same as **c–f**, calculated for zero doping where no distinct surface states are observed. **h**,**i**, Dispersion for propagating (Im *k*_*z*_ = 0, black lines) and evanescent modes (Im *k*_*z*_ ≠ 0, orange lines) for bulk graphite as a function of complex *k*_*z*_ for fixed *ħνk* = 0.04 eV (panel **h**, 1D metal regime with Type 2 evanescent modes) and 0.15 eV (panel **i**, 1D semiconductor regime with Type 1 evanescent modes), respectively. Fermi level is at 0 eV. Green/blue arrows indicate evolution of surface states from propagating modes into evanescent modes for electron/hole doping.

Hexagonal graphite (Bernal stacking) is a compensated semimetal with the Fermi surface occupying only a small fraction of the Brillouin zone. The size of the Fermi surface is determined mostly by a through-layer hopping parameter, *γ*_2_ ≈ −20 meV (ref. ^29^). Owing to its semimetallic nature, graphite does not host surface states (evanescent modes) in the absence of dangling bonds or applied electric field. However, if an electric field above a certain value is applied perpendicular to the basal plane, tunable surface states emerge^26,30^ (see Methods, ‘Surface states in non-aligned graphite films in a zero-*B* field’ and Extended Data Fig. 1).

**Extended Data Fig. 2:**
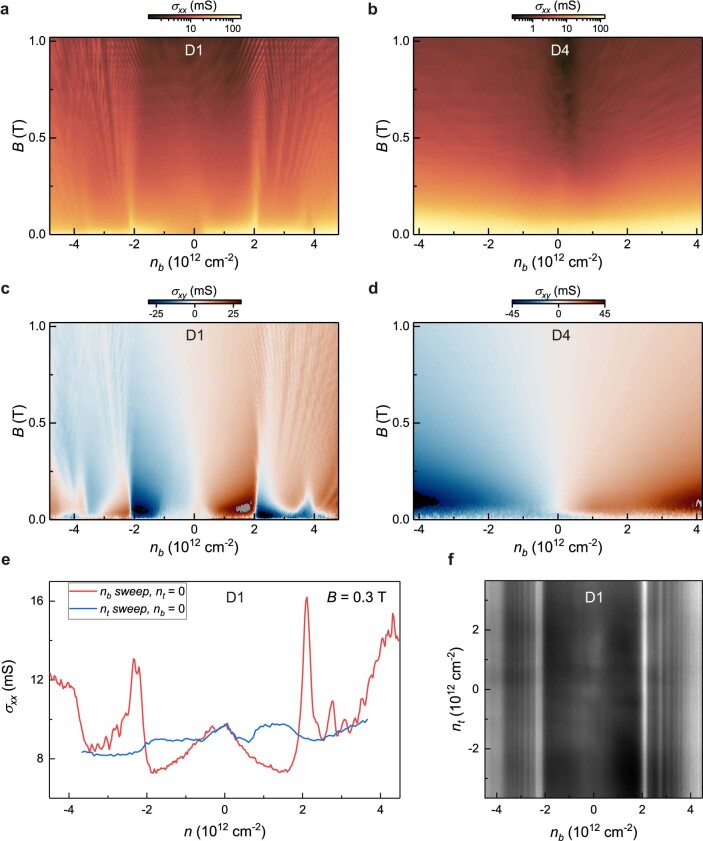
Transport in aligned vs non-aligned interfaces at small magnetic fields. **a**, **c**, *σ*_*xx*_,*σ*_*xy*_ as a function of *B* and *n*_*b*_ for aligned device D1 at *T* = 0.24 K. Landau level features emanate from LTs (|*n*| ≈ 2.0 and 3.7 × 10^12^ cm^−2^) and from zero doping. **b**, **d**, *σ*_*xx*_,*σ*_*xy*_ as a function of *B* and *n*_*b*_ for non-aligned device D4 at *T* = 0.22 K. **e**, Comparison of low field *σ*_*xx*_ line traces for the two interfaces of the single aligned D1, where aligned interface (*n*_*b*_) hosts many features related to the LTs which are notably absent in non-aligned interface (*n*_*t*_) response. **f**, Mapping of *σ*_*xx*_ for D1 as a function of *n*_*b*_ and *n*_*t*_, highlighting that the vertical features related to LTs are independent of *n*_*t*_ throughout the measured range. *B* = 0.3 T, *T* = 1.8 K, and the colour scale is black to white, 5 to 18 mS.

We find that a moiré superlattice at the surface of graphite markedly modifies its surface states, resulting in an entirely different transport behaviour observed between aligned and non-aligned devices (Fig. 1d). Devices with a non-aligned interface show a nearly featureless carrier-density dependence of longitudinal *σ*_*xx*_(*n*) and transversal *σ*_*xy*_(*n*) conductivities in small *B*. By contrast, for the aligned graphite interface, *σ*_*xy*_(*n*) shows multiple zero crossings that are accompanied by peaks in *σ*_*xx*_(*n*). We attribute this behaviour to the recurrence of electrostatically induced surface states occupied by electron- or hole-like charge carriers. To quantify this, we calculated Fermi-surface projections using an effective-mass model with Slonczewski–Weiss–McClure parameterization of graphite^31,32^ subjected to moiré superlattice potential, in combination with self-consistent Hartree analysis (see Methods, ‘Surface states in graphite films in the presence of a moiré superlattice’). Our calculations in the superlattice Brillouin zone (SBZ) of an hBN/graphite/hBN heterostructure show a multitude of surface states with numerous topological Lifshitz transitions (LTs) across a range of carrier densities (Fig. 1e). The four pairs of plots (labelled A–D in Fig. 1e) with considerable changes in the Fermi-surface topology demonstrate four LTs that correspond to the four *n* ranges (Fig. 1d). LTs observed at |*n*| ≈ 2.0 and 3.7 × 10^12^ cm^−2^ belong to two different branches of the surface states—one residing mostly on the first graphene bilayer of graphite and the other mostly on the second bilayer (Extended Data Fig. 1c–f). As *B* increases, the surface states in the vicinity of LTs give rise to separate branches of Landau levels. For details of evolution of *σ*_*xx*_(*n*) and *σ*_*xy*_(*n*) in low magnetic fields see Methods, ‘Surface states in graphite films in the presence of a moiré superlattice’ and Extended Data Fig. 2) Extended Data Fig. 2e,f provides a further comparison of aligned and non-aligned interfaces of device D1, confirming the absence of LTs in surface states at the non-aligned interface.

**Fig. 2:**
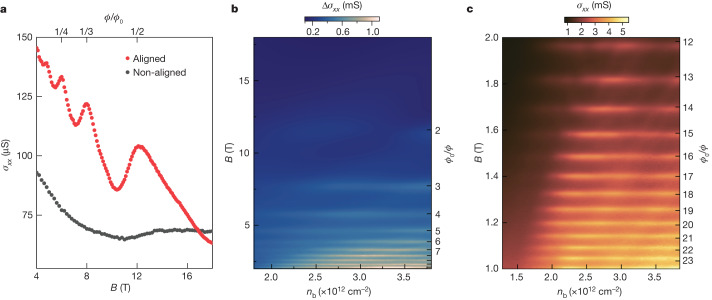
Brown**–**Zak oscillations arising from surface states at the graphite–hBN interface. **a**, Conductivity *σ*_*xx*_ as a function of *B* for device D2 at high electron concentrations induced by the top and bottom gates that dope the aligned and non-aligned graphite–hBN interfaces, respectively. For doping of the aligned interface, peaks appear at values of *B* equivalent to one flux quantum per *q* superlattice unit cells. *n*_t_ = 3.1 × 10^12^ cm^−2^ and *n*_b_ = 3.1 × 10^12^ cm^−2^ for aligned and non-aligned interfaces, respectively. *T* = 60 K. **b**, Conductivity map (a smooth background subtracted, see Methods, ‘Surface states in graphite films in the presence of a moiré superlattice’) as a function of *B* and *n*_b_ at the aligned graphite–hBN interface of device D1. Measurements were performed at 60 K to suppress Landau quantization. The right *y* axis denotes the inverse flux *ϕ*_0_/*ϕ*. **c**, *σ*_*xx*_(*n*_b_,*B*) map for the same device at 20 K.

**Extended Data Fig. 3:**
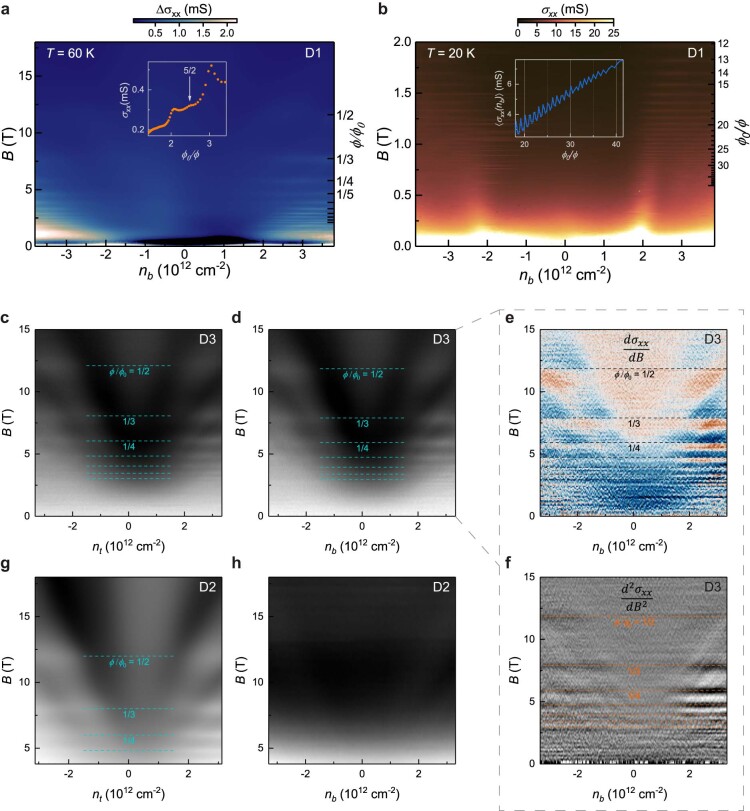
Brown-Zak mappings in aligned devices. **a**, Extended range plot of Fig. 2b in the main text for device D1; *∆σ*_*xx*_ (conductivity minus a smooth background) as a function of *B* and *n*_*b*_ at T = 60 K. Inset, *σ*_*xx*_(*ϕ*_*0*_/*ϕ*) trace at *n*_*b*_ = 3.53 × 10^12^ cm^−2^ highlighting higher order Brown-Zak oscillation at *ϕ*_0_/*ϕ* = 5/2. **b**, Extended range plot of Fig. 2c in the main text; *σ*_*xx*_ as a function of *B* and *n*_*b*_ at *T* = 20 K. Inset, conductivity averaged across a carrier density range *n*_*b*_ = 2.00 to 3.84 × 10^12^ cm^−2^, ‹*σ*_*xx*_(*n*_*b*_)›, plotted as a function of *ϕ*_0_/*ϕ* showing oscillations continuing down to experimental mapping resolution (*B*_*step*_ = 1 mT). **c–h** High temperature mappings of *σ*_*xx*_(*n*_*t*_, *B*) (panels **c** & **g**) and *σ*_*xx*_(*n*_*b*_, *B*) (panels **d** & **h**) for doubly aligned device D3 (**c**–**d**) and singly aligned device D2 (**g**–**h**). Measurements were conducted at *T* = 60 K, colour scale black to white is 80 to 190 µS for **c** & **d**, 49 to 154 µS for **g** and 64 to 97 µS for **h**. Panels **e** & **f** show 1^st^ and 2^nd^ derivatives, respectively, calculated from **d**. Horizontal dashed lines show the significant flux fractions $\phi /{\phi }_{0}$ from 1/2 to 1/8 fitted to the data. Colour scale for **e** is blue to red −40 to 40 µS T^−1^ and in **f** is black to white −100 to 100 µS T^−2^.

With high *B*, the difference between hBN/graphite/hBN devices with aligned and non-aligned interfaces becomes even more prominent (Fig. 2a). The curves *σ*_*xx*_(*B*) were measured at 60 K to suppress Landau quantization. If the aligned surface is doped away from the electron–hole compensation, *σ*_*xx*_ shows an oscillatory behaviour periodic in 1/*B*. Peaks in *σ*_*xx*_ appear in fields ${B}_{1/q}=\frac{1}{q}\frac{{\phi }_{0}}{{A}_{0}}$, corresponding to the integer number *q* of superlattice unit cells with an area *A*_0_ = √3/2*λ*^2^ that are commensurate with the magnetic flux quantum *ϕ*_0_ = *h*/*e*, where *λ* is the wavelength of moiré superlattice, *h* is the Planck’s constant and *e* is the elementary charge. The commensurability between *ϕ*_0_ and the magnetic flux through a moiré unit cell, *ϕ* = *BA*_0_, can be interpreted as a manifestation of Brown–Zak quantum oscillations at the superlattice interface, which were recently reported for aligned monolayer graphene/hBN heterostructures^21,22^. The formation of magnetic Bloch states leads to higher conductivity because of the straight rather than cyclotron trajectories of the surface-charge carriers^21,22,33,34^, as evidenced by the conductivity peaks at *B*_1/*q*_ (Fig. 2a). Figure 2b shows some of these *n*_b_-independent conductivity peaks that were found at all distinguishable 1/*q*-commensurate fields. Note that not only unit fractions but also second-order fractal states (for example, *B*_2/5_ in Extended Data Fig. 3a) can be seen in Brown–Zak oscillations.

**Extended Data Fig. 4:**
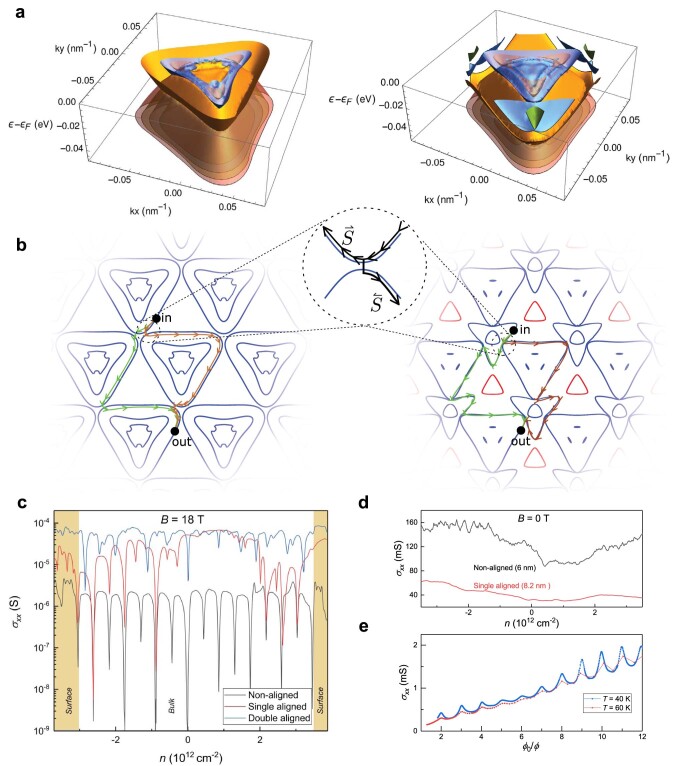
Classical interpretation of Brown-Zak oscillations and conductivity enhancement. **a**, Dispersion in the SBZ plotted up to Fermi energy for doping 2.1 × 10^12^ cm^*−*2^ (left) and 3.8 × 10^12^ cm^*−*2^ (right) respectively. **b**, networks of classical trajectories accessible to surface state electrons at the same dopings in **a**. Examples of shortest interfering paths of equal length are shown by green and brown arrows. The area enclosed between these paths equals *A*_*BZ*_/(*eB*)^2^ irrespective of energy. **c**, Conductivity enhancement at high *B* field in aligned Corbino devices; *σ*_*xx*_ as a function of single gate induced carrier density for non-aligned device (D10, black curve, bottom gate tuned), singly aligned device (D2, red curve, top gate tuned) and doubly aligned device (D3, blue curve, bottom gate tuned) all in Corbino geometry, measured at *T* = 0.3 K and *B* = 18 T. Shaded regions highlight surface Landau band features. **d**, Conductivity at zero *B* field in aligned (D1) vs non-aligned (D9) Hall bar devices. Increased scattering due to alignment results in reduced conductivity in the bulk at *B* = 0 T. Here the top (non-aligned interface) gate is swept for D1. *T* = 0.3 K. **e**, Brown-Zak oscillations in *σ*_*xx*_ differ in amplitude non-monotonically as a function of *ϕ*_0_/*ϕ* in aligned Hall bar device D1. Measured at *n*_*b*_ = 2.6 × 10^12^ cm^−2^, where *T* = 60 K curve is from the same dataset used to generate Fig. 2b and Extended Data Fig. 2a.

Because Brown–Zak oscillations stem from the translational invariance of magnetic Bloch states at rational fractions of magnetic flux *ϕ*/*ϕ*_0_ = *p*/*q* (where *p* is an integer), they are insensitive to temperature as long as electrons retain phase coherence in the area of the magnetic supercell *qA*_0_. Figure 2c shows that at intermediate temperatures (*T* = 20 K), states with *ϕ*_0_/*ϕ* up to 24 are visible (and even states with *ϕ*_0_/*ϕ* > 35 are distinguishable in Extended Data Fig. 3b). This provides a lower bound on the phase coherence length of greater than about 100 nm. Brown–Zak oscillations can also be interpreted as Aharonov–Bohm interference in a periodic 2D network formed by classic trajectories of electrons drifting around the Fermi contours that are joined by magnetic breakdown tunnelling in the vicinity of Van Hove singularities (see Methods, ‘Conventional interpretation of Brown–Zak oscillations’ and Extended Data Fig. 4). This interpretation enables a convenient conceptual transition into the regime of low*-B* fields in which we see multiple LTs of the Fermi-surface topology (Fig. 1e) and explains the disappearance of Brown–Zak oscillations for |*n*_b_| < 2 × 10^12^ cm^−2^.

In comparison, no LTs or Brown–Zak oscillations could be observed in our hBN/graphite/hBN devices if non-aligned interfaces were gated (Figs. 1d and 2a and Extended Data Fig. 2). This is not surprising, as it has previously been shown that states at the opposite surfaces of a graphite film are well screened from each other, with a screening depth of only two to three layers^26^. Raman measurements also do not show any qualitative difference in strain distribution or other effects of alignment for films thicker than seven to eight graphene layers at both aligned and non-aligned graphite interfaces (see Methods, ‘Raman spectroscopy of aligned graphite films’ and Extended Data Fig. 10). This conclusion is further supported by a recent report on atomic relaxation in multilayer moiré heterostructures^23^ that predicts a very short (one layer) penetration depth for moiré reconstruction with superlattice periodicity *λ* < 20 nm.

**Fig. 3:**
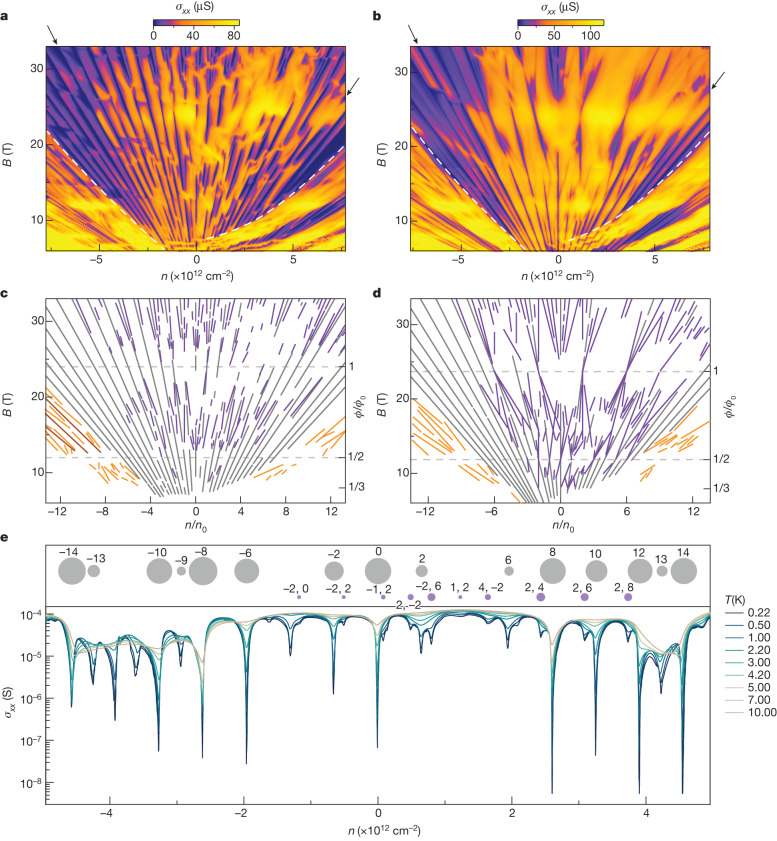
Fractal 2.5D QHE states in graphite. **a**,**b**, Conductivity *σ*_*xx*_ as a function of *n* = *n*_t_ + *n*_b_ and *B* for devices D2 (**a**) and D3 (**b**), *T* = 30 mK and *n*_t_ = *n*_b_ (that is, zero displacement field). The white dashed curves indicate the transition from surface Landau levels to the bulk UQR. Black arrows point to the threshold filling factors that bound the region of bulk in which fractal states are observed (*ν* = −9 and 12). **c**,**d**, Associated Wannier diagrams for panels **a** (**c**) and **b** (**d**): 2D QHE (grey) and fractal 2.5D QHE (purple) states in the UQR. The *x* axis is in units of *n*_0_ = 1/*A*_0_. Below UQR, orange lines trace fractal states and brown lines trace non-fractal states in the surface Landau levels +2 and −2. **e**, Hierarchy of 2.5D QHE gaps in aligned hBN/graphite/hBN. Bottom, *σ*_*xx*_(*n*) traces at different *T* for device D3 at *B* = 13.5 T, which are used to extract gap sizes from Arrhenius activation. Top, bubble plot of the QHE gaps in which the area of circles scales linearly with the found gap sizes (ranging from 30 μeV to 1.8 meV). Grey and purple colour coding is the same as in **d** and labels are integers *s* and *t* from equation (1) for standard QHE (*t* only) and fractal QHE (*s*, *t*) states.

Surprisingly, if the aligned devices are cooled down to our lowest *T* of 30 mK and Landau fan maps are measured, we observe the development of Hofstadter’s butterfly—the fractal QHE—not just in the near-surface 2D states but across the entire graphite film (Fig. 3) as witnessed by gating either bottom (non-aligned) or top (aligned) interface. A high-*B* map of conductivity *σ*_*xx*_ versus *n* = *n*_t_ + *n*_b_ for device D2 (Fig. 3a) shows multiple QHE features. Figure 3c traces the observed conductivity minima on a Wannier diagram. An analogous map and Wannier diagram are presented in Fig. 3b,d for device D3. Although QHE is forbidden in 3D electronic systems, it has recently been reported for thin (up to 100 nm) graphite films^26^. Two main factors contribute to the observed QHE: dimensional reduction of the electronic system from a 3D semimetal to one-dimensional (1D) Landau bands in strong *B* and the consequent formation of standing waves in the 1D Landau bands because of a finite thickness of graphite films. Standing waves result in the quantization of the 1D Landau bands and the development of minigaps, which manifest themselves in a so-called 2.5D QHE. At high fields (above *B* marked by white dashed lines in Fig. 3a,b), only the two lowest Landau bands (0 and 1) cross the Fermi level and contribute to magnetotransport. In addition to being gapped by the standing waves, these two bands are split by an energy gap *δ*_10_ ≈ 0.4 meV T^−1^, and further spin-resolved by the Zeeman gap, 2*μ*_B_*B* (*μ*_B_ is the Bohr magneton). Lifting the +KH and −KH valley degeneracy of these bands depends on the graphite layer parity^26^.

**Extended Data Fig. 5:**
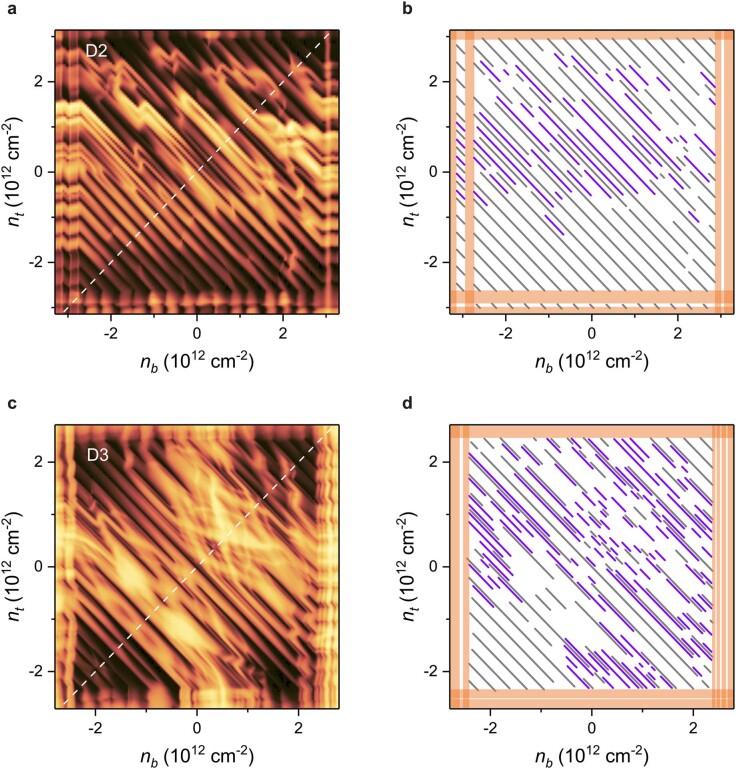
Existence of fractal states throughout the bulk. **a**, *σ*_*xx*_(*n*_*t*_, *n*_*b*_) of device D2 as a function of top and bottom gates, *n*_*b*_ and *n*_*t*_, measured at high field *B* = 15.6 T, *T* = 0.3 K, colour scale: brown to yellow, 0 to 59 μS. **b**, Wannier diagram depicting the QHE and fractal QHE gaps in the bulk as diagonal grey and purple lines, respectively. High doping for top (bottom) gate results in horizontal (vertical) features that correspond to surface states accessing the +2 and −2 Landau bands from the opposite graphite surfaces (highlighted by orange shading). **c**, *σ*_*xx*_(*n*_*t*_, *n*_*b*_) of device D3, measured at high field B = 13.6 T, T = 0.3 K, colour scale: brown to yellow, 0 to 98 μS. **d**, The same as **b** but for D3.

To show how the QHE states of Hofstadter’s butterfly penetrate through the entire graphite bulk, we have also measured *σ*_*xx*_ as a function of *n* using both top and bottom gates at fixed *B* (see Extended Data Fig. 5a,c and the corresponding Wannier diagrams in Extended Data Fig. 5b,d). 2.5D QHE gaps appear as diagonal features because these Landau levels can be filled equivalently by either *n*_b_ or *n*_t_ and, therefore, the states extend throughout the bulk. This is the case for both standard QHE and Hofstadter’s butterfly gaps, which shows that, in the ultraquantum regime (UQR), the moiré surface potential affects the entire bulk of graphite. Conductivity maps *σ*_*xx*_(*n*_t_, *n*_b_) at the high*-B* field for doubly aligned device D3 is generally similar to that of singly aligned device D2, with QHE and Hofstadter’s butterfly gaps following both *n*_t_ and *n*_b_ (Extended Data Fig. 5). One notable difference attributed to the symmetry breaking of the interface alignment is that *σ*_*xx*_(*n*_t_, *n*_b_) is asymmetric for singly aligned device D2, but symmetric for doubly aligned device D3 (Extended Data Fig. 5).

**Fig. 4:**
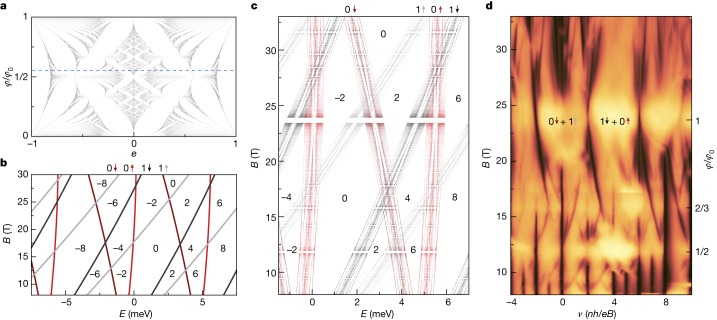
Hofstadter broadening of energy levels in graphite. **a**, Hofstadter’s butterfly calculated for a honeycomb lattice following ref. ^36^, with a normalized energy scale. The dashed line marks *ϕ*/*ϕ*_0_ equivalent to *B* = 13.5 T, the field strength as in Fig. 3e. **b**, Landau levels resulting from quantized states from 0 Landau bands are shown in red and 1 Landau bands are shown in grey and calculated for a 16-layer-thick graphite film without a moiré perturbation^26^. Zeeman splitting is included, as indicated by lighter and darker curves for the spin up and down, respectively. Labels in black refer to the filling factor *ν*. **c**, Expected spectra by applying Hofstadter’s butterfly in **a** as a small perturbation to each Landau level in **b**. Same labelling as in **b**. **d**, Conductivity map replotted from Fig. 3b as a function of *ν*. Two prominent gap closures around *ϕ*/*ϕ*_0_ = 1 have been labelled by the Landau band origin and spin of the levels that are crossing; the same colour coding as in **b**,**c**. Colour scale: brown to yellow, 0 μS to 115 μS.

Hofstadter’s butterfly^35^—a fractal set of energy eigenvalues for magnetic fluxes *ϕ*/*ϕ*_0_ = *p*/*q*—is shown in Fig. 4a for a honeycomb lattice^36^, matching the geometry of our moiré superlattice. In electron transport measurements, this fractal pattern manifests itself in a Landau fan diagram and its Wannier representation, which are described by the Diophantine equation:

1 $$\frac{n}{{n}_{0}}=t\frac{\phi }{{\phi }_{0}}+s,$$

where integer *t* is the Landau filling factor *ν* = *nh*/*eB* and integer *s* is the superlattice Bloch band-filling index; *n*_0_ = 1/*A*_0_ is the density of one electron per superlattice unit cell. For *s* = 0, equation (1) corresponds to the conventional Landau fan with *t* ≡ *ν* (Fig. 3c,d, grey lines), whereas for *s* ≠ 0, it traces Hofstadter states (Fig. 3c,d, purple lines) emanating from magnetic fields satisfying *ϕ*/*ϕ*_0_ = *p*/*q*.

Figure 3e shows a hierarchy in the observed QHE gaps: those measured at integer filling factors (*s* = 0) are one order of magnitude larger than the gaps because of Hofstadter’s butterfly (*s* ≠ 0). This suggests that the effect of the moiré superlattice on the QHE can be considered a small perturbation. To model the impact of this perturbation, we envelop the standing waves of 0 and 1 Landau bands in graphite with Hofstadter’s butterfly energy spectrum. Figure 4b shows the Landau-level spectrum calculated for a 16-layer-thick graphite film without taking into account moiré perturbation (see Methods, ‘Bulk states in graphite films in the presence of a surface moiré superlattice’), where Landau levels cross each other with increasing *B*, which corresponds to closure and subsequent reopening of gaps in the 2.5D QHE. Figure 4c plots the same 16-layer-graphite spectrum but each Landau level, *E*_m_, is now augmented by the Hofstadter’s butterfly spectrum, *ε*, using

2 $${E}_{{\rm{m}}}^{{\rm{moir \acute{{\rm{e}}} }}}={E}_{{\rm{m}}}+S\varepsilon .$$

Here *S* ≈ 0.42 meV is the scaling factor for the bandwidth of Hofstadter’s butterfly^37^, which was estimated from the measured transport gaps. The obtained ${E}_{{\rm{m}}}^{{\rm{moir \acute{{\rm{e}}} }}}$ spectrum shows good agreement with our experimental data in terms of both sizes and positions of the gaps (Fig. 4d) (gap closures at *ϕ*/*ϕ*_0_ = 1 are labelled with crossings of the corresponding ${E}_{{\rm{m}}}^{{\rm{moir \acute{{\rm{e}}} }}}$ states).

Bulk fractal states observed here are different from those of graphene-based 2D electronic systems, and our mixed moiré system demonstrates plenty of additional, non-trivial physics, inaccessible in 2D systems. First, the conductivity of bulk graphite can be efficiently tuned using interface alignment—*σ*_*xx*_ is increased more than two times at zero *B* field and up to an order of magnitude at high *B*  in aligned versus non-aligned devices (Extended Data Fig. 4c,d). Second, *B*-field dependence of the amplitude of Brown–Zak oscillations observed in aligned graphite films differs from that of graphene, showing a non-monotonic dependence of the amplitude of oscillations on 1/*B* and emphasizing the richness of our 3D twistronics system (Extended Data Fig. 4e). Third, the absence of bulk fractal states beyond threshold filling factors (*ν* = −9 and 12; Fig. 3a,b and Extended Data Fig. 5) shows that the mixing of moiré surface and bulk states can be controlled electrostatically by changing the screening of moiré potential to the bulk bands.

In summary, we have shown that surface states in graphite (and, potentially, other semimetals and doped semiconductors) can be strongly modified by a moiré superlattice potential. The alignment between hBN and graphite provides a kaleidoscope of LTs that develop into Brown–Zak oscillations and Hofstadter surface states. Remarkably, moiré surface states in high magnetic fields also affect the entire electronic spectrum of these graphite films, which results in fractal Hofstadter butterflies that can be referred to as 2.5D in analogy with the 2.5D QHE in graphite. Our approach thus offers a possibility to explore mixed-dimensionality effects arising because of surface superlattices extending their influence deep into the bulk of 3D electronic systems.

## Methods

### Device fabrication

To make hBN/graphite/hBN heterostructures, graphite flakes were encapsulated by hBN through dry transfer as described elsewhere^38,39^. In brief, graphite and hBN flakes were mechanically exfoliated onto oxidized silicon substrates. The target hBN flake was picked up by a polymer film made of polydimethylsiloxane and polymethylmethacrylate and then used to pick up a graphite flake of known thickness. The obtained stack is then released onto another hBN flake on an SiO_2_/Si wafer, completing the heterostructure. To make aligned hBN/graphite structures, the straight edges of hBN and graphite flakes, which are usually along their crystallographic axes, were aligned in parallel.

Top-gate electrode and metal contacts to graphite (3 nm Cr/80 nm Au) were patterned using electron beam (e-beam) lithography and reactive ion etching, followed by an e-beam evaporation process. These devices were then shaped into Hall bar geometry using a thermally evaporated aluminium film as etch mask, which was later removed by 0.1 M NaOH solution. Alternatively, for Corbino devices, we utilized e-beam overexposure of polymethylmethacrylate resist to form a crosslinked bridge, which separates the inner contact, the top gate and the outer contact.

Graphite capacitor devices used to study surface states of non-aligned heterostructures were fabricated similarly, with the hBN flake intentionally misaligned to the underlying graphite film on a quartz substrate. A quartz substrate was chosen to minimize the parasitic capacitance, known to be a feature of SiO_2_/Si substrates. Graphite flakes of around 50 nm thickness were used, guaranteeing the 3D graphite electronic spectrum. Relatively thick hBN flakes (>40 nm) were also chosen to eliminate the inhomogeneity of electrostatic potential introduced by a relatively rough metal electrode.

### Transport and capacitance measurements

The longitudinal and Hall voltages of Hall bar devices were recorded with lock-in amplifiers (SR830 or MFLI) on applying a small low-frequency a.c. current of 10 nA (except where a higher current is specified). For Corbino devices, a small ac bias (40–100 μV) was applied to the inner contact, and the current was recorded from the outer one using SR830 in current input mode (lock-in amplifier input resistance of 1 kΩ and any in-line filters were subtracted from the measured resistance to account for any voltage drop across these components). The conductivity of Corbino devices was calculated using *σ*_*xx*_ = 1/(2*π*)ln(*r*_o_/*r*_i_)*G*, where *G* is the measured conductance and *r*_o_ is the outer radius and *r*_i_ is the inner radius of the graphite channel. Magnetic fields up to 18 T were generated by superconducting magnets, while data above 18 T were obtained in a 20 MW resistive magnet at the LNCMI-Grenoble.

To confirm the alignment or misalignment of the top and bottom graphite/hBN interfaces and to extract the moiré wavelength *λ*, we used the following two methods: (1) measuring the Landau fan diagrams of surface states at each interface by sweeping either the top or bottom gate voltages, respectively, and (2) measuring high-temperature Brown–Zak oscillations. Because the two surface states are electronically decoupled, they can feel only the potential in the vicinity of the corresponding interface because of electrostatic screening. For doubly aligned device D3, both surface states show Brown–Zak oscillations with conductivity peaks at nearly the same *B* fields, indicating the same moiré period for the top and bottom interfaces. We fitted multiple oscillations corresponding to integer flux fractions *ϕ/ϕ*_0_ from 1/2 to 1/8 to *σ*_*xx*_ and derivatives $\frac{{\rm{d}}{\sigma }_{xx}}{{\rm{d}}B}$ and $\frac{{{\rm{d}}}^{2}{\sigma }_{xx}}{{\rm{d}}{B}^{2}}$ (Extended Data Fig. 3c,d), which yields a value of *B*_0_ for each sequence of oscillations, where *B*_0_ is the magnetic field at which *ϕ*_0_ = *B*_0_*A*_0_ with magnetic flux quantum *ϕ*_0_ and superlattice unit cell area *A*_0_. Using this value of *B*_0_, the moiré wavelength *λ* is calculated as *λ* = $\sqrt{2{A}_{0}/\sqrt{3}}$. Furthermore, *B*_0_ can also be extracted from fractal QHE states fitting at low temperatures. The difference in *λ* between the two interfaces calculated using these two methods was less than or equal to 0.1 nm. Given the similarity in the measured *λ* from Brown–Zak oscillations and the appearance of only one set of fractal states in the dual-gate maps in Fig. 3 (where distinct moiré periods would be expected to generate multiple sets of fractal states), we confirm the alignment of both interfaces with a precision of ±0.1 nm.

Differential capacitance *C* was measured as a function of bias voltage *V*_b_ between the metal gate and graphite using an on-chip cryogenic bridge^40^, which reaches a sensitivity of about 10 aF at 1-mV excitation. Excitations of 102.53-kHz frequency and opposite phases were applied to the sample and a reference capacitor. Output signals from these two capacitors were mixed at the gate of a high-electron-mobility transistor, which served as an amplifier. The excitation voltage of the reference capacitor was modulated so that the output signal from the high-electron-mobility transistor becomes zero, and the capacitance of the sample is obtained from the ratio of excitation voltages at the balance point. A typical excitation voltage applied to the samples ranged from 1 to 10 mV, depending on the thickness of the hBN dielectric layer.

### Surface states in non-aligned graphite films in a zero-*B* field

To compute the surface states, we adapted an effective-mass model of a finite-thickness graphite film using the Slonczewski–Weiss–McClure (SWMC) parameterization^26,31,32^ combined with the self-consistent potential profile of graphite sandwiched between two gates with carrier densities *n*_t_ and *n*_b_. In the Hartree approximation, the potentials on the layers, *U*_*j*_ > 1, are related to layer electronic densities *n*_*l*_ as

3 $${U}_{j}={U}_{1}+\frac{{e}^{2}c}{\varepsilon \,{\varepsilon }_{0}}\left[\left(\,j-1\right){n}_{{\rm{b}}}+\left(\mathop{\sum }\limits_{p=1}^{j-1}\mathop{\sum }\limits_{l=p+1}^{2N}{n}_{l}\right)-\frac{\varepsilon -1}{4}\left({n}_{1}-{n}_{j}\right)\right],$$

where *ε* = 2.6 accounts for the vertical polarizability of graphene^41^, *c* = 3.35 Å is the interlayer separation and 2*N* is the number of graphene layers. We temporarily fix the value of *U*_1_, which has the role of a surface chemical potential and then self-consistently calculate Hartree potentials and densities on all the layers. The electronic density in layer $l$ of graphite, calculated in the Hartree approximation, is

4 $${n}_{l}=2{\int }_{{\rm{BZ}}}\frac{{{\rm{d}}}^{2}{\bf{k}}}{{(2{\rm{\pi }})}^{2}}\mathop{\sum }\limits_{n=1}^{4N}f\left({\varepsilon }_{n}\left({\bf{k}}\right)\right)\left({\left|{\varPsi }_{n}^{{\rm{A}}}\left(l,{\bf{k}}\right)\right|}^{2}+{\left|{\varPsi }_{n}^{{\rm{B}}}\left(l,{\bf{k}}\right)\right|}^{2}\right)-{n}_{0},$$

where *f* is a Fermi–Dirac distribution, and *n* enumerates the eigenfunctions for a given in-plane momentum **k**. The constant *n*_0_ is chosen to match ${\sum }_{l=1}^{2N}{n}_{l}=0$ to provide electrical neutrality. After finding the densities on all the layers, we relate *U*_1_ to *n*_t_ using ${n}_{{\rm{t}}}=-{n}_{{\rm{b}}}-\,{\sum }_{n=1}^{2N}{n}_{l}$.

To examine the thermodynamic density of states (DOS) at the graphite–hBN interface, we use capacitance spectroscopy, which is a tool that has been applied to 2D systems^40,42^. However, its application to study surface states of metals or semimetals is rare. The measured capacitance (*C*) can be considered as geometric parallel-plate capacitance *C*_G_ = *εε*_0_*A*/*d* and quantum capacitance *C*_Q_ in series, 1/*C* = 1/*C*_G_ + 1/*C*_Q_, where *A* is the device area, *ε*_0_ is the vacuum permittivity and *d* and *ε* are the thickness and relative permittivity of the hBN dielectric layer^43^. The quantum capacitance reflects the DOS = d*n*/d*U*_1_ on the surface of graphite: *C*_Q_ = *Ae*^2^d*n*/d*U*_1_, where *n* is the carrier density and *e* is the electron charge. In our measurements, *C* follows a V-shaped dependence on *n*, where $n({V}_{{\rm{b}}})=\frac{1}{Ae}{\int }_{0}^{{V}_{{\rm{b}}}}C\left(V\right){\rm{d}}V$, with a notable fine structure (Extended Data Fig. 1a).

The capacitance (per unit area) can be calculated self-consistently from equations (3) and (4) as

5 $${C}^{-1}\left(n\right)={C}_{{\rm{G}}}^{-1}+{\left.{\left(-e\frac{{\rm{d}}n}{{\rm{d}}{U}_{1}}\right)}^{-1}\right|}_{{{U}_{1}=U}_{1}\left(n\right)}.$$

By comparing the calculated capacitance with experimental data, we obtained a set of SWMC parameters (*γ*_0_ = 3.16 eV, *γ*_1_ = 0.39 eV, *γ*_2_ = −17 meV, *γ*_3_ = −0.315 eV, *γ*_4_ = 44 meV, *γ*_5_ = 38 meV and Δ_*AB*_ = 50 meV). Results of this procedure are shown in Extended Data Fig. 1b, showing excellent agreement between theory and experiment. At low doping (|*U*_1_| < |*γ*_2_|, that is, |*n*| < 6 × 10^11^ cm^−2^), there are no surface states, and quantum capacitance plotted in Extended Data Fig. 1b is determined by electron and hole screening. Because holes have a slightly larger DOS (shallower dispersion) than electrons, we see larger *C*_Q_ at hole doping. When doping reaches *U*_1_ ≈ ±*γ*_2_, type 1 and type 2 surface states appear and contribute to the quantum capacitance. The radius of the surface Fermi line for type 1 states grows with |*n*|, leading to an increase in the density of surface states and growth of *C*_Q_. Examples of the graphite film dispersion spectra with self-consistently determined layer potentials are shown in Extended Data Fig. 1c–g for *n* ranging from −6 ×  10^12^  cm^−2^ to 6 × 10^12^ cm^−2^, where the red colour coding represents a high probability for wavefunctions at the first graphene bilayer and the green colour coding represents a high probability for wavefunctions at the second graphene bilayer.

To provide a qualitative understanding of the surface states in graphite, we analytically solve the spectrum of graphite^32^, considering the boundary conditions (*Ψ* = 0 for surface carbon atoms) and plot the eigenstates for homogeneous bulk graphite (Extended Data Fig. 1h,i), which consist of a propagating mode (black curves, real *k*_*z*_) and an evanescent mode (orange curves, complex *k*_*z*_). There are no complex *k*_*z*_ solutions at zero doping, as only real *k*_*z*_ solutions satisfying zero boundary conditions can be normalized. Electrostatic doping of graphite surface creates an inhomogeneous *z* direction potential near the surface, which does not preserve *k*_*z*_ momentum, allowing real *k*_*z*_ solutions near the surface, which then turn into evanescent modes decaying into the bulk. This provides a heuristic picture of the origin of non-trivial surface-state solutions (Extended Data Fig. 1h,i).

There are three types of propagating mode: majority electron and hole bands with bandwidth 2*γ*_2_ and a minority carrier band near *ck*_*z*_ = π/2. These propagating bands cross the bulk Fermi level at a small in-plane momentum *k*_*x*_,*k*_*y*_ (Extended Data Fig. 1h, 1D metal regime in the *z* direction) but are spread away from the Fermi level at large *k*_*x*_,*k*_*y*_ (Extended Data Fig. 1i, 1D semiconductor regime in the *z* direction). When a potential near the surface is introduced by doping, the propagating modes in the 1D semiconductor region start to cross the Fermi level. With potential abating away from the surface, these modes evolve into evanescent modes in the gap (Extended Data Fig. 1i, green arrows for electron doping and blue arrows for hole doping). The dispersion of these evanescent modes, which we denote as type 1, crosses the Fermi level, forming a surface Fermi line with a radius larger than the Fermi surface of propagating carriers (Extended Data Fig. 1c–g, yellow contours). These states are similar to surface states in doped semiconductors, with the difference that they exist for only in-plane momenta larger than the projection of the bulk Fermi surface of graphite (no surface states observed for zero doping in Extended Data Fig. 1g). In the 1D metal regime, another type of evanescent mode, denoted as type 2, appears for |*E*| > |*γ*_2_| and never crosses the Fermi level (Extended Data Fig. 1h).

### Surface states in graphite films in the presence of a moiré superlattice

The spectrum for graphite aligned with hBN was calculated by treating the periodic moiré potential as a perturbation applied to only the top graphene layer. We followed the standard procedure^19^, using the mirror-symmetric superlattice coupling Hamiltonian $\delta H={\sum }_{m=0}^{5}{{\rm{e}}}^{{{\rm{i}}{\bf{g}}}_{m}\cdot {\bf{r}}}\,\left[{U}_{0}+{(-1)}^{m}({\rm{i}}\,{U}_{3}{\sigma }_{3}+{U}_{1}\frac{{{\bf{g}}}_{m}\times \hat{z}}{|g|}\sigma ){\tau }_{3}\right]$ applied to the two top-layer components of the graphite film wavefunction, where Pauli matrices *σ* operate on top-layer sublattices and *τ* operates on valleys, ${{\bf{g}}}_{m}={{\mathcal{R}}}_{\pi (m-1)/3}\{0,\,4\pi \delta /(3a)\}$ are six reciprocal lattice vectors of superlattice (where ${\mathcal{R}}$ is a rotation matrix), $\delta =0.018$ is a lattice mismatch, *a* = 1.42 Å is carbon–carbon distance, and we use the parameters *U*_0_ = 8.5 meV, *U*_1_ = −17 meV and *U*_3_ = −14.7 meV (refs. ^44,45^). The results do not significantly depend on the values of superlattice couplings, and it was sufficient to restrict the momentum space to the first star of the superlattice reciprocal lattice vectors to achieve convergence.

At low fields (*B* < 1 T), the onset of 2.5D QHE is strongly altered by the kaleidoscopic band structure of the surface states (Fig. 1e). We compare the low field transport for aligned (D1) and non-aligned (D4) devices of similar graphite thickness (approximately 8  nm) (Extended Data Fig. 2a–d). In a non-aligned graphite device, we observe that a Landau fan develops for finite densities |*n*_b_| > 10^12^ cm^−2^, and all QHE states can be traced back to *n*_b_ ≈ 0 as *B* approaches 0. By contrast, for aligned graphite similar QHE features are also overlaid by oscillations emanating from LTs at |*n*| ≈ 2.0 and 3.7 × 10^12^ cm^−2^ resulting in the diamond-like features in *σ*_*xx*_ occurring at flux fractions *ϕ*/*ϕ*_*0*_ = *p*/*q*. Comparison of low field conductivity as a function of tuning aligned and non-aligned interfaces in the same device also shows pronounced differences, as shown in Extended Data Fig. 2e,f, where the most visible features occur only at |*n*_b_| > 2 × 10^12^ cm^−2^, independent of *n*_t_ doping.

To highlight Brown–Zak oscillations across a large range of magnetic fields, we also calculated Δ*σ*_*xx*_ by subtracting a smooth background from the *σ*_*xx*_ data. In comparison to graphene–hBN systems in which the background conductivity can be fitted with polynomials^21^, we find that even-higher-order (>10) polynomials are insufficient as many oscillatory artefacts are present. Instead, we use a two-carrier Drude model of *σ*_*xx*_(*B*) and *σ*_*xy*_(*B*) and fit both simultaneously to yield carrier densities and mobilities *n*_h_ = 2.2 × 10^12^ cm^−2^, *µ*_h_ = 24,000 cm^2^ V^−1^ s^−1^, *n*_e_ = 2.8 × 10^12^ cm^−2^ and *µ*_e_ = −19,000 cm^2^ V^−1^ s^−1^ for zero gate bias at *T* = 60 K. This two-carrier model fit, ${\sigma }_{xx}^{{\rm{fit}}}(B)$, is then used to calculate ${\Delta \sigma }_{xx}\left({n}_{{\rm{b}}},B\right)={\sigma }_{xx}\left({n}_{{\rm{b}}},B\right)-{\sigma }_{xx}^{{\rm{fit}}}\left(B\right)$. Oscillations in Δ*σ*_*xx*_ occurring at ${B}_{1/q}$ visible for *q* ≤ 11 (Fig. 2b and Extended Data Fig. 3a) were cross-examined against raw *σ*_*xx*_ data to confirm they were not introduced by the subtraction process.

### Bulk states in graphite films in the presence of a surface moiré superlattice

To model the transport gaps in our aligned devices at high *B* and low *T*, we treat moiré superlattice potential as a weak perturbation; each 2.5D QHE Landau level (Fig. 4b) is split into *q* subbands, at a given *ϕ*/*ϕ*_0_ = *p*/*q*. Levels in Fig. 4b were calculated from the tight-binding description of Landau bands in graphite from ref. ^26^ using the same set of SWMC parameters as stated above, with an adjustment to the splitting between Landau bands 0 and 1 attributed to the effects of self-energy in high*-B* fields^46,47^. Hofstadter’s butterfly for a honeycomb lattice (Fig. 4a) was calculated from the finite-difference equation in ref. ^36^, in which the energy scale has been normalized, and is given in arbitrary units, *ε* = ±1. A limit of *q* ≤ 100 was used for the computation to give a balance between plot density and speed. However, this results in the apparent absence of states near *ϕ*/*ϕ*_0_ = 1, 1/2 and 1/3 (Fig. 4a,c), and it should be noted that this is a feature of the computation, not gaps in the spectra.

Analysis of the thermal activation of gaps for device D3 at *B* = 13.5 T (Fig. 3e) indicates the largest fractal gaps are Δ*E*_fractal_ ≈ 0.1 meV. We assign Δ*E*_fractal_ to the largest gap in Hofstadter’s butterfly at the flux value *ϕ*/*ϕ*_0_ = 0.57 (corresponding to *B* = 13.5 T; Fig. 4a, dashed line), which spans 0.32 < *ε* < 0.56. This yields a scaling factor *S* = 0.42 meV. The full spectrum is then calculated using equation (2) and shown in Fig. 4c, in which we use the periodicity of Hofstadter’s butterfly (such that *ε*(*ϕ*/*ϕ*_0_ + *ρ*) = *ε*(*ϕ*/*ϕ*_0_) for any integer *ρ*) to plot states at *ϕ*/*ϕ*_0_ > 1.

**Extended Data Fig. 6:**
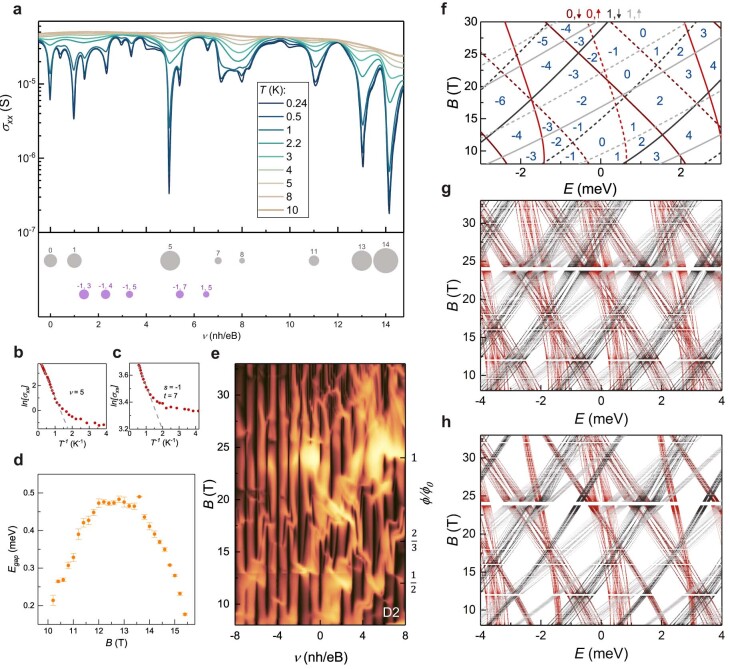
Gap size hierarchy in device D2. **a**, Upper panel, σ_xx_ as a function of integer QHE filling factor ν at various temperatures, B = 15.2 T. Lower panel, bubble plot of extracted gap size from Arrhenius fits to the σ_xx_ minima. Gap size scales with area (60 µeV to 0.9 meV), and grey bubbles are integer gaps (labelled by ν) and purple bubbles are fractal gaps (labelled by s, t). **b**,**c**, Examples of Arrhenius plots of ln[σ_xx_] as a function of reciprocal temperature for integer QHE gap at ν = 5 and fractal QHE gap of integers (s,t) = (−1,7), respectively. The linear regions are fitted to yield a slope of ≈ ½E_gap_. **d**, Gap size extracted from Arrhenius fits as a function of B for the ν = 0 gap. **e**, Conductivity map for device D2, same data as in Fig. 3a in the main text, except plotted as a function of filling factor ν of main QHE sequence. Colour scale: brown to yellow, 0 to 80 μS. **f**, Allowed energy levels resulting from quantised states from 0 (1) Landau bands shown in red (grey), calculated for 21-layer-thick graphite film without a moiré perturbation^26^. Zeeman splitting has been included, as indicated by light and dark lines for spin up and spin down, respectively. **g**, Combination of panels **f** and Fig. 4a in the main text by applying Hofstadter’s butterfly as a small perturbation (S = 0.42 meV) to each energy level, Eq. 2. **h**, Same as **g** but with S = 0.42 meV for odd layer states and S = 0.12 meV for even layer states.

For comparison, the fractal energy spectrum was also computed for device D2, which has a different alignment to hBN and layer parity to that of device D3 (device D2 is 21 layers in thickness and aligned to only one encapsulating hBN). Odd-layer parity lifts the ±KH valley degeneracy in 2.5D QHE in graphite^26^ and, therefore, the gap size is significantly reduced (Extended Data Fig. 6a–c) and the maximal gap size is about 0.9 meV (compared with 1.8 meV in device D3; Fig. 3e). In Extended Data Fig. 6d, we focus on the evolution of gap size at filling *ν* = 0 between two level crossings at *B* = 10 T and *B* = 16 T, with a maximal observed gap of about 0.48 meV. Extended Data Fig. 6f shows the Landau levels without moiré perturbation for the 21-layer graphite. Both the extent of the *ν* = 0 gap (8.5 T < *B* < 17 T) and its maximal size (1.3 meV) in the model are notably larger than those observed in the experiment. On application of Hofstadter’s butterfly to each Landau level (using the same *S* = 0.42 meV as in Fig. 3e), each level has effectively broadened and thus the *ν* = 0 gap in the model is reduced to about 0.6 meV (Extended Data Fig. 6g), in closer agreement with our experiments. However, the broadening of energy levels from Hofstadter’s butterfly leads to many overlapping states and hence gap closures, which were not observed in our experiment. This is probably because of inadequate treatment of moiré perturbation to states hosted on even and odd layers. As the moiré reconstruction is limited to a very short penetration depth, the perturbation will be much larger on the outermost layer (odd) than on subsequent layers. We plot a revised model with *S* = 0.42 meV for odd layers and *S* = 0.12 meV for even layers (Extended Data Fig. 6h), yielding fewer gap closures, whereas the same *ν* = 0 gap remains, which is in overall better agreement with our experiment (Extended Data Fig. 6e).

### Surface states in non-aligned graphite films in finite *B* fields

**Extended Data Fig. 7:**
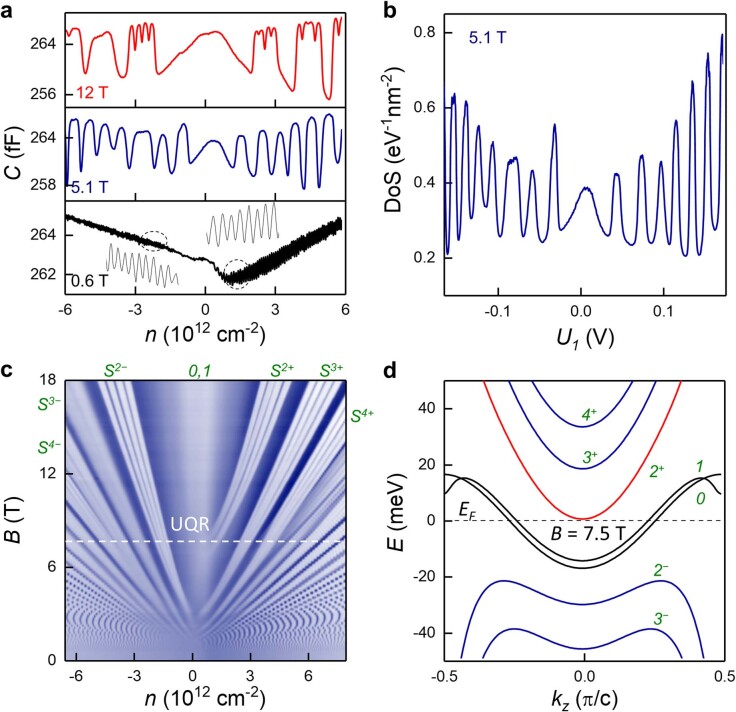
Magnetocapacitance oscillations and Landau fan diagram of the surface states. **a**, Typical *C*(*n*) at 0.6 T (bottom black curve), 5.1 T (middle blue curve) and 12 T (top red curve), respectively, in capacitor device D6 at *T* = 0.3 K. Bottom insets are magnifications of the marked areas. **b**, *DoS vs U*_1_ at 5.1 T, 0.3 K. **c**, Surface Landau fan diagram *C* (*n*, *B*) at 0.3 K. Colour scale: navy to white, 254.7 fF to 270.0 fF. Dashed line marks the critical field *B* = 7.5 T, where bulk Landau band 2^+^ leaves the Fermi level and graphite enters the UQR. **d**, Dispersion relation for bulk Landau bands calculated using the SWMC model at *B* = 7.5 T^26^.

**Extended Data Fig. 8:**
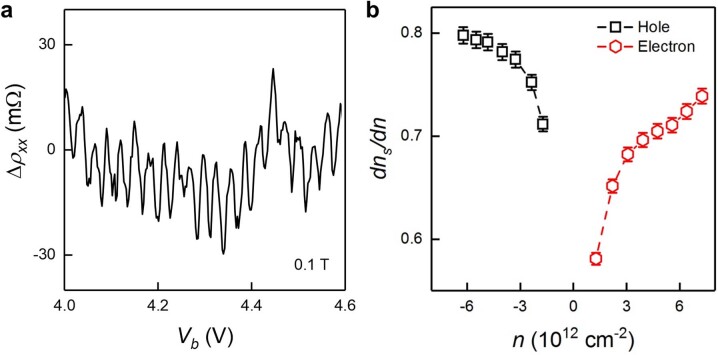
Low field quantum oscillations. **a**, Oscillations of Δ*ρ*_*xx*_ in device D7 obtained by subtracting a smooth background while sweeping the gate voltage (*V*_*b*_) at 0.1 T, 0.3 K using excitation *I* = 1 μA. The encapsulated graphite with a thickness of 20 nm is defined to Hall bar geometry for this measurement. **b**, *dn*_*s*_/*dn* as a function of *n* at 0.6 T, where *n*_*s*_ is the carrier density injected into the surface states, *n* is the total electrostatically induced carrier density. It is deduced from the curve shown in Extended Data Fig. 7a, based on the periodicity of the oscillations.

In the *B* field, surface states manifest in the capacitance spectra as pronounced magnetocapacitance oscillations (Extended Data Fig. 7a). For bulk Landau bands that cross the Fermi level, the associated surface states would coexist and mix with them. However, bulk Landau bands away from the Fermi level can become occupied at the surface when electrostatically doped, giving rise to surface Landau levels. On filling these surface Landau levels, regions of high compressibility appear as peaks in the capacitance spectra. Note that the width of these high-compressibility regions does not correspond to integer degeneracy (>4), because some fraction of gate-voltage-induced charge is sunk into the bulk to support the self-consistent screening potential near the surface (Extended Data Fig. 8b).

Having determined the geometric capacitance from the fitting of zero field data, we can convert *C*(*n*) into DOS(*U*_1_) using *U*_1_ = *eV*_b_ − *e*^2^*n*/*C*_G_ (ref. ^40^). As shown in Extended Data Fig. 7b, peaks in the DOS correspond to metallic-surface Landau levels, which are separated by relatively low DOS regions (cyclotron gaps of the surface states). In contrast to true 2D systems, the DOS in these cyclotron gaps is non-zero, because charges can be injected into the bulk graphite. At 12 T, three minima are further developed on top of most peaks, indicating that the fourfold degeneracy (spin and valley) of the surface Landau levels is lifted.

Experimental results are better visualized and more informative when presented as a *C*(*n*, *B*) map (Extended Data Fig. 7c). The branches of surface states spawn out from the neutrality points at *B* = 7.5 T, 3 T, 2 T and so on. These *B* fields correspond to the critical fields above which the bulk Landau bands no longer cross the Fermi level and appear as only surface Landau levels. For instance, according to our SWMC model, at 7.5 T, the bulk Landau band 2^+^ is just above the Fermi level (Extended Data Fig. 7d). Thus, a branch of surface states spawned out around this field is labelled as *S*^2+^. The same happens with the electron bulk Landau band 3^+^ at 3 T and hole bulk Landau band 2^−^ at 2 T (Extended Data Fig. 7c).

**Extended Data Fig. 9:**
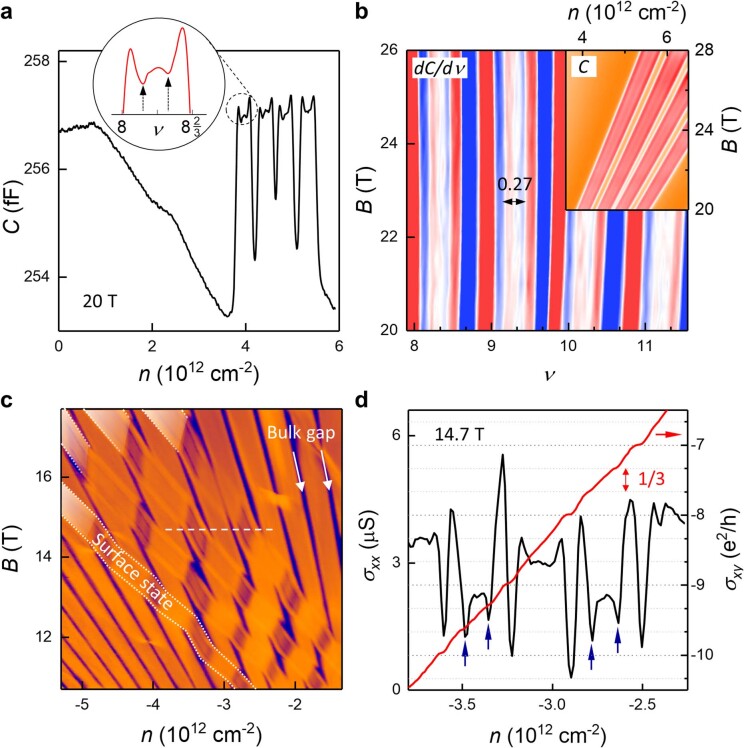
Fractional surface states in non-aligned graphite. **a,b**, Fractional surface states measured in capacitance (device D8). **a**, *C*(*n*) at *B* = 20 T, *T* = 0.3 K. Inset magnifies the encircled region, but plots as *C*(*ν*). **b**, *dC/dν* (*ν, B*) of S^2+^ in high *B* region. Colour scale: blue to red, −6 to 6 fF. Right-top inset shows the corresponding *C* (*n, B*) map. Colour scale: orange to red, 254.3 to 260 fF. **c**, Longitudinal conductivity *σ*_*xx*_ (*B*, *n*) measured at *D* = 0.24 V/nm in a Hall bar device D9, *T* = 0.3 K, *I* = 20 nA. The white shaded areas are guides to the eye for the surface states. Boundaries of one such surface states are marked by white dotted curves. Logarithmic colour scale: navy to orange, 0.1 to 118.2 µS. **d**, *σ*_*xx*_ cut profile (black curve) of the white dashed line in **c** and the corresponding Hall conductivity *σ*_*xy*_ (red curve).

We observed oscillations down to *B* ≈ 0.1 T (Extended Data Fig. 8a), which sets a lower bound of approximately 100,000  cm^2^  V^−1^  s^−1^ for surface-charge carrier mobility. The high electronic quality of surface states also enables fractional features in the Landau quantization of charge carriers. A graphite capacitor device was fabricated to investigate fractional QHE features, with a thicker hBN dielectric to reduce the inhomogeneity of electrostatic potential from the metal electrode. At a high magnetic field, *B* = 20 T, we observe the formation of two minima on top of singly degenerate surface states of *S*^2+^ (Extended Data Fig. 9a,b). The Δ*ν* between the fractional gap is around 0.27, which is lower than the expected Δ*ν* = 1/3 for fractional QHE. To further investigate these fractional QHE states, we used thin (6 nm) graphite (device D9) and studied transport under an applied displacement field, *D* = (*n*_t_ − *n*_b_)*e*/2*ε*_0_. At *D* = 0.24 V nm^−1^, *B*–*n* regions can be found in which the energy level of surface states locates in the bulk gap (Extended Data Fig. 9c,d). In these regions, the surface states are isolated from the bulk completely, and vanishing *σ*_*xx*_ and quantized *σ*_*xy*_ indicate the development of fractional QHE with a 1/3 degeneracy. The difference between the capacitance and transport measurements can be reconciled by considering the negative compressibility of the fractional states: the chemical potential of the surface states reduces with the injection of *n*, acquiring additional charges from the bulk^48–50^.

### Conventional interpretation of Brown–Zak oscillations

The classical dynamics of the electron is set by $\dot{{\bf{p}}}=eB\hat{{\bf{z}}}{\boldsymbol{\times }}\dot{{\bf{r}}}$ and $\dot{{\bf{r}}}\equiv {\bf{v}}={{\nabla }}_{{\bf{p}}}\varepsilon ({\bf{p}})$, which implies that the real-space trajectories can be obtained from constant energy contours in momentum space by a 90° rotation and rescaling by 1/*eB*. Near Van Hove singularities, caused by the saddle points in the dispersion of electrons, the change of the sign of the band mass occurs, which is known as the LT. At the LT, closed cyclotron orbits of electrons transform into open trajectories, forming a network, which, because of the *C*_3_ symmetry of graphite film, looks like a Kagome pattern. This leads to delocalized electron orbits resulting in high conductivity even at strong magnetic fields, even though the electron ballistic motion along such a network has a stochastic element: when reaching the saddle points in dispersion, electron paths can switch between electron- and hole-like segments (Extended Data Fig. 4a,b). This process, known as the magnetic breakdown of cyclotron motion, can be captured^51–54^ by transmission amplitudes, $\mathop{S}\limits^{\leftharpoonup }$ and $\mathop{S}\limits^{\rightharpoonup }$,

$$\frac{| \mathop{S\,}\limits^{\rightharpoonup }| }{| \mathop{S\,}\limits^{\leftharpoonup }| }=\exp \,\left[\frac{{\rm{\pi }}\hbar (\varepsilon -{E}_{{\rm{LT}}})}{eB{\mathfrak{r}}}\right];\,| \mathop{S\,}\limits^{\rightharpoonup }{| }^{2}+| \mathop{S\,}\limits^{\leftharpoonup }{| }^{2}=1.$$

These magnitudes of $| \mathop{S}\limits^{\rightharpoonup }|$ and $| \mathop{S}\limits^{\leftharpoonup }|$ are comparable to each other in the magnetic breakdown^55^ interval of energies, proportional to $eB{\mathfrak{r}}/(2\pi \hbar )$, which is determined by the strength of *B* field and Gaussian curvature of the dispersion saddle point, ${\mathfrak{r}}=\hbar \sqrt{| \det \frac{{\partial }^{2}\varepsilon ({\rm{p}})}{\partial {p}_{i}\partial {p}_{j}}| }$ and sets the energy window around *E*_LT_, where the LT network of trajectories is relevant for electron transport.

For any pair of points in the network, there are several distinct equal-length paths connecting them. These paths consist of an equivalent set of segments passed in a different order (for example, green and brown paths in Extended Data Fig. 4b), which—because of the periodicity of the Kagome network—ensures independence of the interference phase between partial waves following those paths, on the exact energy of electrons (similar to the physics of weak localization). As a result, broadening of the Fermi step does not lead to self-averaging of constructive and destructive interference contributions generated by electrons at various energies (as happens with the interference-induced mesoscopic fluctuations). The length of each segment of the trajectory scales as 1/*B*. So, for low *B*, only the shortest possible trajectories retain ballistic propagation (see Extended Data Fig. 4b for examples of such pairs of trajectories). The area, $A=\frac{{A}_{{\rm{BZ}}}}{{(eB)}^{2}}=\frac{{(2{\rm{\pi }})}^{2}}{{{A}_{0}(eB)}^{2}}$, between the pairs of such trajectories is related—by rescaling with *B* field—to the actual Brillouin zone area, ${A}_{{\rm{BZ}}}={(2{\rm{\pi }})}^{2}/{A}_{0}$, of the superlattice, where ${A}_{0}$ is the unit supercell area. Multiplied by the magnetic field, this determines the encircled magnetic flux $\phi ={A}_{0}B$ and the Aharonov–Bohm phase, $\varphi =\hbar eAB=\hbar \frac{{(2{\rm{\pi }})}^{2}}{{A}_{0}eB}=2{\rm{\pi }}\frac{{\phi }_{0}}{\phi }$. The interference between partial waves that undergo ballistic propagation along the Kagome trajectories and stochastic switching at the Kagome network sites produce conductivity oscillations,

6 $$\Delta \sigma \propto \frac{B}{\delta n}{{\rm{e}}}^{-\frac{{\left(n-{n}_{{\rm{LT}}}\right)}^{2}}{\delta {n}^{2}}}{{\rm{e}}}^{-\frac{2{\mathcal{L}}}{{\mathcal{l}}}}\cos \left(2{\rm{\pi }}\frac{{\phi }_{0}}{\phi }\right),$$

$$\delta n=2\rho \sqrt{{({k}_{{\rm{B}}}T)}^{2}+\frac{2}{5}{\left(\frac{B{\mathfrak{r}}}{{\phi }_{0}}\right)}^{2}},$$

which are 1/*B* periodic. At low magnetic fields, the length of the paths, ${\mathcal{L}}(B)\propto {B}^{-1}$, would be longer than the shortest of the mean free path and coherence lengths, ${\mathcal{l}}$, which is captured by the exponential factor in equation (6). Here *ρ* is the thermodynamic density of states and the width, $\delta n$, of the doping interval around LT in which the oscillations are expected to be visible is determined by both *T* and *B*. Note that the described oscillations are related to the ability of the electron to propagate across the superlattice rather than its density of states. Thus, they appear in the conductivity measurements but would be absent in quantum capacitance measurements. We also note that for a graphite surface aligned with hBN, LTs start to appear in surface and mixed bulk-surface bands after surface doping reaches 2 × 10^12^ cm^*−*2^. With increasing doping, there is a cascade of LTs (Extended Data Fig. 4a,b). As a result, the interval of densities, in which the above-described 1/*B*-periodic oscillations are visible, is broadened.

The mean free path, ${\mathcal{l}}$, appearing in the denominator of the exponent of our expression for the amplitude of oscillations in equation (6) is usually assumed to depend on only the temperature, and equation (6) produces an exponential decay, ${\rm{\exp }}\left(-\frac{2{\mathcal{L}}}{{\mathcal{l}}}\right)$, of oscillations at low magnetic fields. However, in the case of graphite, there are a lot of Fermi contours of bulk bands nearer to the *K* point and the increasing magnetic field could result in magnetic breakdown scattering from the surface to the bulk bands. This additional scattering decreases the lifetime of electrons on surface-state Fermi contours, effectively decreasing the mean free path, ${\mathcal{l}}$, with growing *B*. This mechanism may lead to non-monotonic dependence of the amplitude of oscillations on 1/*B* (Extended Data Fig. 4e), reflecting the complexity of our 3D twistronics system.

### Raman spectroscopy of aligned graphite films

**Extended Data Fig. 10:**
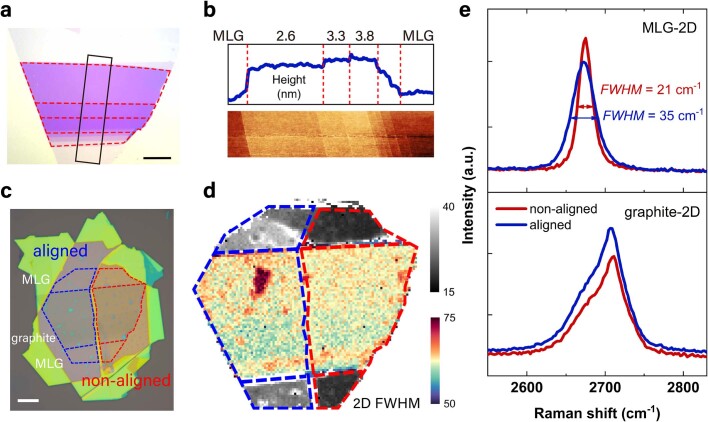
Raman characterization of aligned and non-aligned ABA graphite. **a**, Optical image and **b**, AFM profile of graphite flake used in Raman measurement (area shown in the black box in **a**). The flake contains both regions of MLG and graphite with thickness around 10 layers. Scale bar 10 um. **c**, Optical image of the stack fabricated for Raman measurements. The aligned and non-aligned regions are marked by blue and red dashed lines. Scale bar 10 um. **d**, Raman map of full width half maximum (FWHM) of 2D peak. The MLG and graphite regions are colour-coded in grayscale and red-to-purple, respectively. **e**, Comparison of 2D peak between aligned and non-aligned regions in MLG (up) and graphite (down). Each spectrum shown here is averaged over ten spectra at different spots. The intensity is normalized by that of hBN peak at 1363 cm^−1^.

To characterize the effect of surface superlattice potential on hBN-encapsulated graphite, we performed Raman spectroscopy measurements. A graphite flake with an extended monolayer graphene (MLG) region was selected to benchmark the alignment of the entire graphite film (Extended Data Fig. 10a,b). Raman spectra of MLG/hBN superlattices have been well studied^56^, and the alignment can be traced by the width of the 2D peak of MLG. The 2D peak of MLG broadens with better alignment because of the increased strain inhomogeneity caused by the moiré periodic potential of the hBN substrate. Similar broadening of the 2D peak was also observed in the bilayer graphene–hBN superlattice system^57^, indicating that the superlattice potential of the hBN substrate can propagate through graphene bilayers, and is therefore detectable by Raman spectroscopy. However, how far this superlattice potential can penetrate the bulk of graphite remains unclear.

To clarify this, we fabricated two hBN/graphite/hBN heterostructures at the same time, by transferring graphite onto two adjacent but intentionally misoriented hBN flakes. The graphite flake is controlled to be aligned with one of the hBNs, and as a consequence is misaligned with the other (Extended Data Fig. 10c). The flake alignment is characterized by the full width at half maximum (FWHM) of the MLG 2D peak (Extended Data Fig. 10e). Each spectrum was averaged over ten spectra acquired at different positions and normalized by the intensity of the *E*_2g_ hBN peak at 1,363 cm^−1^. The FWHM is 21 cm^−1^ and 35 cm^−1^ for non-aligned and aligned regions of the MLG, respectively, which agrees well with the results in ref. ^56^. Broadening of the 2D peak is expected if the superlattice potential at the interface can propagate through the bulk graphite crystal. We found no appreciable difference on the Raman map of 2D FWHM between aligned and non-aligned graphite regions (Extended Data Fig. 10d,e). This implies that the surface superlattice potential of the hBN substrate does not penetrate through graphite, at least for films of thickness at least 2.6 nm.

## Online content

Any methods, additional references, Nature Portfolio reporting summaries, source data, extended data, supplementary information, acknowledgements, peer review information; details of author contributions and competing interests; and statements of data and code availability are available at 10.1038/s41586-023-06264-5.

## Figures and Tables

**Extended Data Table 1:**
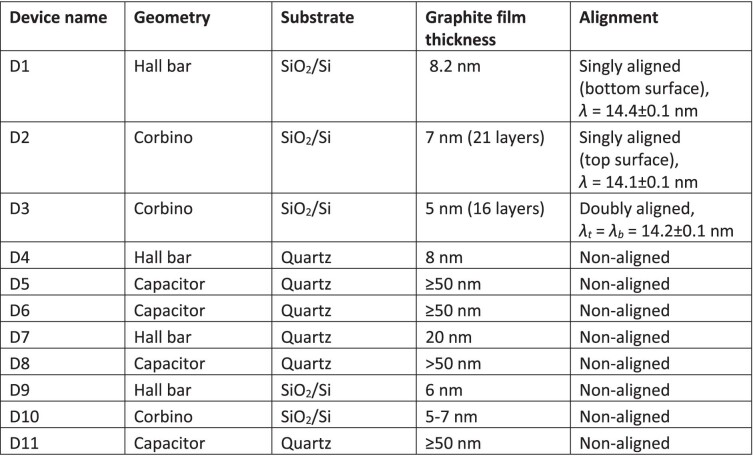
Details of the studied devices

## Data Availability

All data are available from the corresponding authors upon reasonable request.

## Code availability

The codes generated in this work to compute surface states of graphite multilayers with and without coupling to aligned hBN are available at https://github.com/slizovskiy/GraphitehBN.

## References

[1] Mak KF, Shan J (2022). Semiconductor moiré materials. Nat. Nanotechnol..

[2] Lau CN, Bockrath MW, Mak KF, Zhang F (2022). Reproducibility in the fabrication and physics of moire materials. Nature.

[3] Ciarrocchi A, Tagarelli F, Avsar A, Kis A (2022). Excitonic devices with van der Waals heterostructures: valleytronics meets twistronics. Nat. Rev. Mater..

[4] Liu Y (2021). Moiré superlattices and related moiré excitons in twisted van der Waals heterostructures. Chem. Soc. Rev..

[5] Kennes DM (2021). Moiré heterostructures as a condensed-matter quantum simulator. Nat. Phys..

[6] He F (2021). Moiré patterns in 2D materials: a review. ACS Nano.

[7] Carr S, Fang S, Kaxiras E (2020). Electronic-structure methods for twisted moiré layers. Nat. Rev. Mater..

[8] Yankowitz M, Ma Q, Jarillo-Herrero P, LeRoy BJ (2019). van der Waals heterostructures combining graphene and hexagonal boron nitride. Nat. Rev. Phys..

[9] Andrei EY (2021). The marvels of moiré materials. Nat. Rev. Mater..

[10] Cao Y (2018). Correlated insulator behaviour at half-filling in magic-angle graphene superlattices. Nature.

[11] Cao Y (2018). Unconventional superconductivity in magic-angle graphene superlattices. Nature.

[12] Chen, G. et al. Tunable correlated Chern insulator and ferromagnetism in a moiré superlattice. *Nature* **579**, 56–61 (2020). 10.1038/s41586-020-2049-7 32132694

[13] Alexeev EM (2019). Resonantly hybridized excitons in moiré superlattices in van der Waals heterostructures. Nature.

[14] Regan EC (2020). Mott and generalized Wigner crystal states in WSe_2_/WS_2_ moiré superlattices. Nature.

[15] Li H (2021). Imaging two-dimensional generalized Wigner crystals. Nature.

[16] Xu Y (2020). Correlated insulating states at fractional fillings of moiré superlattices. Nature.

[17] Liu E (2021). Signatures of moiré trions in WSe_2_/MoSe_2_ heterobilayers. Nature.

[18] Zhou Y (2021). Bilayer Wigner crystals in a transition metal dichalcogenide heterostructure. Nature.

[19] Ponomarenko LA (2013). Cloning of Dirac fermions in graphene superlattices. Nature.

[20] Hunt B (2013). Massive Dirac fermions and Hofstadter butterfly in a van der Waals heterostructure. Science.

[21] Krishna Kumar R (2017). High-temperature quantum oscillations caused by recurring Bloch states in graphene superlattices. Science.

[22] Krishna Kumar R (2018). High-order fractal states in graphene superlattices. Proc. Natl Acad. Sci. USA.

[23] Halbertal, D. et al. Multilayered atomic relaxation in van der Waals heterostructures. *Phys. Rev. X.* **13**, 011026 (2023).

[24] Mandelli D, Ouyang W, Urbakh M, Hod O (2019). The princess and the nanoscale pea: long-range penetration of surface distortions into layered materials stacks. ACS Nano.

[25] Davison, S. G. & Steslicka, M. *Basic Theory of Surface States* (Oxford Univ. Press, 1996).

[26] Yin J (2019). Dimensional reduction, quantum Hall effect and layer parity in graphite films. Nat. Phys..

[27] Shi Y (2020). Electronic phase separation in multilayer rhombohedral graphite. Nature.

[28] Yang Y (2019). Stacking order in graphite films controlled by van der Waals technology. Nano Lett..

[29] Dresselhaus MS, Dresselhaus G (2002). Intercalation compounds of graphite. Adv. Phys..

[30] Morozov SV (2005). Two-dimensional electron and hole gases at the surface of graphite. Phys. Rev. B.

[31] Slonczewski JC, Weiss PR (1958). Band structure of graphite. Phys. Rev..

[32] McClure JW (1957). Band structure of graphite and de Haas-van Alphen effect. Phys. Rev..

[33] Brown E (1964). Bloch electrons in a uniform magnetic field. Phys. Rev..

[34] Zak J (1964). Magnetic translation group. Phys. Rev..

[35] Hofstadter DR (1976). Energy levels and wave functions of Bloch electrons in rational and irrational magnetic fields. Phys. Rev..

[36] Rammal R (1985). Landau level spectrum of Bloch electrons in a honeycomb lattice. J. Phys..

[37] Huber R (2022). Band conductivity oscillations in a gate-tunable graphene superlattice. Nat. Commun..

[38] Mishchenko A (2014). Twist-controlled resonant tunnelling in graphene/boron nitride/graphene heterostructures. Nat. Nanotechnol..

[39] Wang L (2013). One-dimensional electrical contact to a two-dimensional material. Science.

[40] Yu GL (2014). Hierarchy of Hofstadter states and replica quantum Hall ferromagnetism in graphene superlattices. Nat. Phys..

[41] Slizovskiy S (2021). Out-of-plane dielectric susceptibility of graphene in twistronic and Bernal bilayers. Nano Lett..

[42] Yu GL (2013). Interaction phenomena in graphene seen through quantum capacitance. Proc. Natl Acad. Sci. USA.

[43] Luryi S (1988). Quantum capacitance devices. Appl. Phys. Lett..

[44] Wallbank JR (2019). Excess resistivity in graphene superlattices caused by umklapp electron–electron scattering. Nat. Phys..

[45] Lee M (2016). Ballistic miniband conduction in a graphene superlattice. Science.

[46] Sugihara K (1984). Charge-density wave and magnetoresistance anomaly in graphite. Phys. Rev. B.

[47] Arnold F (2017). Charge density waves in graphite: towards the magnetic ultraquantum limit. Phys. Rev. Lett..

[48] Eisenstein JP, Pfeiffer LN, West KW (1992). Negative compressibility of interacting two-dimensional electron and quasiparticle gases. Phys. Rev. Lett..

[49] Eisenstein JP, Pfeiffer LN, West KW (1994). Compressibility of the two-dimensional electron gas: measurements of the zero-field exchange energy and fractional quantum Hall gap. Phys. Rev. B.

[50] Li L (2011). Very large capacitance enhancement in a two-dimensional electron system. Science.

[51] Wilkinson M (1997). Critical properties of electron eigenstates in incommensurate systems. Proc. R. Soc. Lond. A.

[52] Cohen MH, Falicov LM (1961). Magnetic breakdown in crystals. Phys. Rev. Lett..

[53] Davis LC, Liu SH (1967). Landau spectrum and line broadening in real metals. Phys. Rev..

[54] Berry MV, Mount KE (1972). Semiclassical approximations in wave mechanics. Rep. Prog. Phys..

[55] Alexandradinata A, Glazman L (2018). Semiclassical theory of Landau levels and magnetic breakdown in topological metals. Phys. Rev. B.

[56] Eckmann A (2013). Raman fingerprint of aligned graphene/h-BN superlattices. Nano Lett..

[57] Cheng B (2015). Raman spectroscopy measurement of bilayer graphene’s twist angle to boron nitride. Appl. Phys. Lett..

